# The Complicated Effects of Extracellular Vesicles and Their Cargos on Embryo Implantation

**DOI:** 10.3389/fendo.2021.681266

**Published:** 2021-06-04

**Authors:** Nan-Xing Jiang, Xue-Lian Li

**Affiliations:** ^1^Obstetrics and Gynecology Hospital, Fudan University, Shanghai, China; ^2^Shanghai Key Laboratory of Female Reproductive Endocrine Related Diseases, Obstetrics and Gynecology Hospital, Fudan University, Shanghai, China

**Keywords:** extracellular vesicles, exosomes, embryo implantation, intercellular communication, microRNA

## Abstract

As a rate-limiting step in pregnancy, embryo implantation is highly dependent on intercellular communication. Extracellular vesicles (EVs) are newly identified to be important in the course of intercellular communication. EVs have been isolated from a wide variety of biofluids and tissues, including plasma, liver, uterine, semen, embryo, etc. The present and future use of EVs not only as biomarkers, but also as targeting drug delivery system, is promisingly pave the way for advanced comprehension of implantation failure in reproductive diseases. However, as the precise mechanisms of EVs in embryo implantation has not been elucidated yet. Herein, we summarize the current knowledge on the diverse effects of EVs from various sources and their cargos such as microRNA, long non-coding RNA, protein, etc. on embryo implantation, and the potential mechanisms of EVs in reproductive diseases such as recurrent implantation failure, polycystic ovary syndrome and endometriosis. It is essential to note that many of the biologically plausible functions of EVs in embryo implantation discussed in present literatures still need further research *in vivo*.

## Introduction

Implantation is a continuous dynamic process during which the blastocyst is implanted in the receptive endometrium in the mid-luteal phase. Embryo implantation is the starting point and a rate-limiting step of pregnancy. The success of embryo implantation mainly depends on two factors: zygote and corresponding establishment of endometrial receptivity. The essential processes of embryo implantation involve “location”, “adhesion” and “invasion”. Endometrial receptivity is present only for a very short time in the mid-secretory phase of each menstrual cycle, typically occurring in the 22th to 24th days of the cycle (1). This fleeting moment is called “windows of implantation (WOI)”.

Contrary to the widespread belief, an article proposed that “embryo implantation” should more properly be regarded as the “war” between embryo and endometrium (2). The embryo uses a variety of coercive tactics to force its acceptance by endometrium. It’s not a cooperation or an accommodation, but an aggression and a conquest. This metaphor is not exaggerated because the natural conception rate of human is very low (only 30-40%) (3). Additionally, 75% of pregnancy losses are due to failed embryo implantation (4). Even with the help of assisted reproductive technology, the pregnancy rate is still low, the culprit remains implantation failure (5, 6). Take polycystic ovary syndrome (PCOS) as an example, even we are able to obtain high-quality oocytes by controlled ovarian hyperstimulation and ideal blastocytes by *in vitro* fertilization, the rate of clinical pregnancy and live birth is still lower in women with PCOS comparing with that of women without PCOS, which is mainly due to the impaired endometrial receptivity (7, 8). At present, embryo implantation has obviously become an unsolved hot issue in the field of reproductive medicine research, and the mechanisms of embryo implantation need further study. Embryo implantation only occurs when the development of embryo coordinate with the station of endometrium, which is highly dependent on intercellular communication (9–11). Intercellular communication in traditional sense relies on intimate physiology contact or soluble mediators in microenvironment such as hormones, growth factors, cytokines, chemokines and proteases (12–16). In recent years, a series of papers have revealed a bran-new communication mechanism that modulates embryo implantation, which is called intercellular communication mediated by extracellular vesicles (EVs) (13, 17, 18).

The aim of this review is to summarize the current knowledge about the physiological roles of EVs produced by maternal tissues, embryo, semen as intercellular messengers in embryo implantation process. We have also reviewed how EVs affect embryo implantation in reproductive diseases.

## Methods

**Figure 1:**
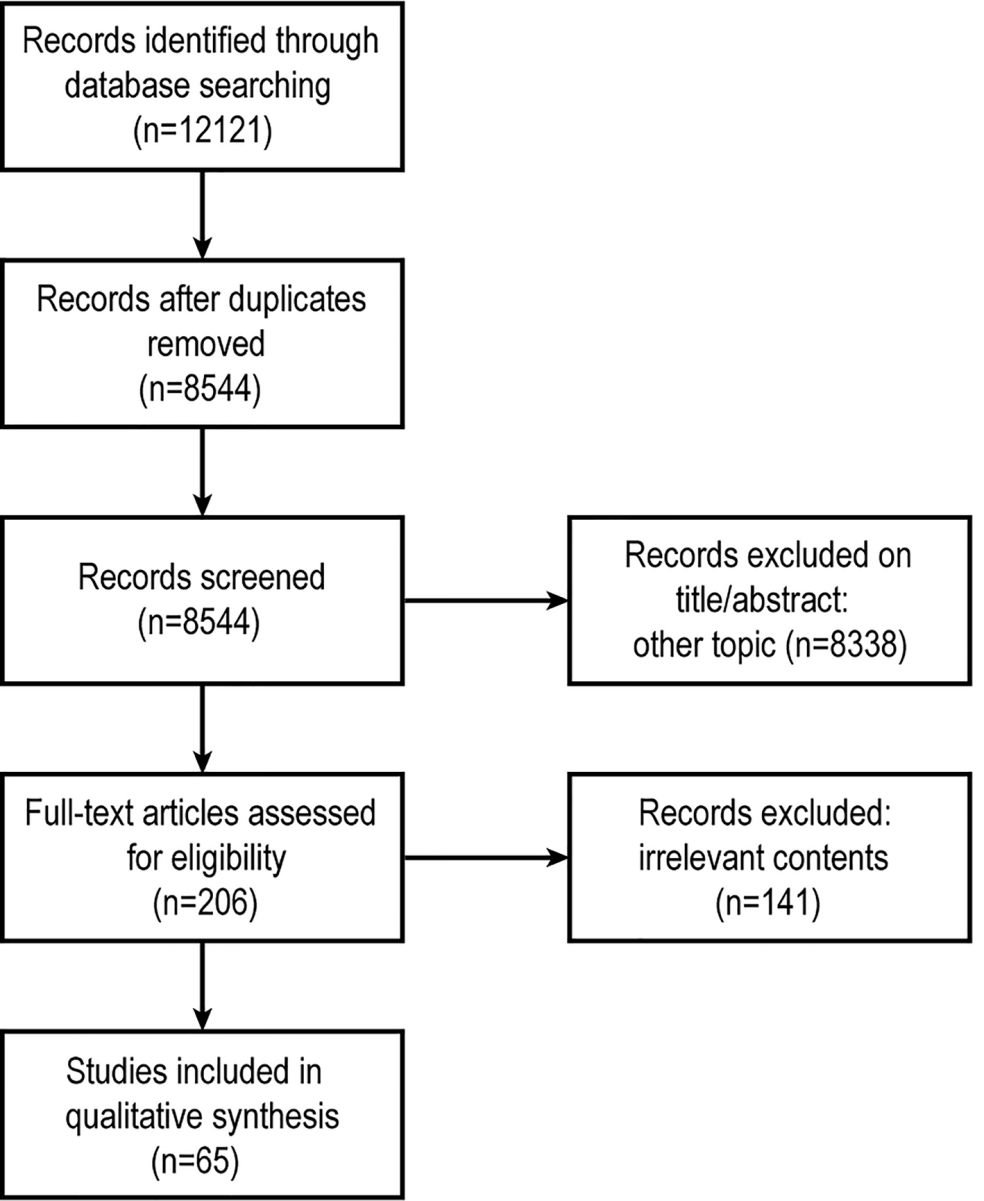
Schematic of study selection.

The present review includes three strategies: literature search, study selection, and results summary. A systematic review was performed using the PubMed, Medline (Ovid), Embase (Ovid), and Web of Science databases without additional limits (**Figure 1**). We used the following query: (‘extracellular vesicles’ or ‘exosomes’ or ‘microparticles’ or ‘microvesicles’) and (‘embryo implantation’ or ‘embryo development’ or ‘endometrium’ or ‘polycystic ovary syndrome’ or ‘recurrent implantation failure’ or ‘endometriosis’). The last search was run on 25 April 2021. Both animal and human studies were considered suitable for this review. Research studies on EVs (“apoptotic bodies” and “apoptotic vesicles” are not included) or their regulation in embryo implantation, discussing either the endometrial receptivity, embryo, or both, were eligible for inclusion.

We identified 12121 articles after a primary search by the databases (**Figure 1**), immediately excluded 3577 records because they were duplicated. Then two Reviewers independently screened articles by title and abstract, any discrepancies were resolved by consensus. 8338 records were excluded (including irrelevant topics, reviews, comments, replies or letters to the Editor). The remaining 206 articles were collected as full-texts. Finally, after full-text screening, 65 articles were used for qualitative analysis.

## Extracellular Vesicles

EVs are cell-derived membranous vesicles without specific targets. The cargos of EVs are heterogeneous, including nucleic acids (DNA, mRNA, microRNAs and long non-coding RNAs), proteins, lipids and so on. Almost all cells can produce and release EVs. Once released into extracellular space, EVs can produce local effects through autocrine and paracrine methods, Or be transported to distant organs or tissues through body fluids such as blood and lymph as important carriers for molecular exchange between different kinds of cells in short or long distance (19). However, there is still a lack of consensus on the nomenclature of EVs (19). We propose to distinguish three different types of EVs on the basis of biogenetic pathway and physical characteristics: 1. exosomes, 2. microparticles (MPs) (or microvesicles, MVs) and 3. apoptotic bodies (17, 20). Exosomes are 40-120 nm size homogenous vesicles, which originated from multivesicular bodies (21, 22) and enriched in major histocompatibility complex class I (MHC class I), MHC class II and tetraspanins such as CD6, CD9, CD63, CD81, as well as protein markers like Alix, TSG101 and chaperones (23, 24).

## EVs and Embryo Implantation

Embryo implantation is divided into three steps (25): (1) Establishment of endometrial receptivity (2), Endometrial decidualization regulated by embryonic signals (3), Trophoblast invasion. Production of EVs by various tissues/cells such as endometrium, decidua, embryo, seminal fluid, oviduct as well as diverse stem cells has been reported to have an impact on the three steps of embryo implantation (summarized in **Table 1**). There are 30 EVs-related references in Section 4.

**Table 1:** Summary of key characteristics of EVs and their implication in embryo implantation.

Source of EVs	Markers	Targets	Functions	References
**Endometrium**	CD9, CD63, ALIX, TGS101	Embryo and trophoblast cells	Increase adhesion, invasion and regulate embryo energy	(26–31)
**DSC**	CD9, CD63, ALIX	Trophoblast cells	Induce trophoblast cells invasion	(32)
**Embryo**	CD9and TGS101,	Embryo and endometrium	Improve preimplantation embryonic development	(21)
			Alter the expression of specific transcripts in endometrium	(33)
**ES**	flotillin-2 (MV)	Embryo and trophoblast cells	Stimulate trophoblast migration	(18)
**Semen**	CD63, CD81	Endometrial stromal cells	Regulate endometrial immuno-inflammatory responses	(34–36)
			Promote the prolactin secretion and enhance decidualization of eSCs	
**Oviduct**	CD9	Embryo	Decrease apoptosis of embryonic cells	(37, 38)
			Improve the mitochondrial heath of embryo	(39)
			Enhance embryonic development by regulating ROS and 5-mC levels	(40)
**EndMSC**	CD9, CD63	Embryo and endometrium	Increase the quality of the embryo	(41, 42)
			ROS elimination and immunoregulation in embryo	(41)
			Angiogenesis, differentiation and tissue remodeling of the endometrium	(42, 43)
**ADSC**	Alix, CD63	Endometrium	Promote endometrial angiogenesis	(44)
			Regulate the expression of molecular markers related to endometrial receptivity	
**BMSC**	CD9, HSP70	Endometrium	Promote endometrial repair by the TGF-β1/Smad signaling pathway	(45)
**UC-MSC**	CD63, TSG101	Endometrium	Promote endometrial regeneration and fertility recovery through immunomodulation	(46, 47)

### Endometrium-Derived EVs

Endometrial epithelial cell-derived exosomes can fuse with developing embryos and functionally affect the process of adhesion and invasion of blastocysts (26, 27, 48, 49), which partially mediated by active focal adhesion kinase (FAK) signaling (28, 29). Endometrial epithelial cell-derived exosomes can enhance human trophectodermal spheroid adhesion and outgrowth capacity (30). Besides, endometrial epithelial-exosomes-treated embryos have an increased implantation rate *in vivo* (30). Exosomal microRNAs from endometrial epithelial cells, including miR-218, ensure trophoblast cell development by targeting sFRP2 and regulating the WNT signaling pathway under conditions of endometritis (31). In addition, Embryo ATP production can be modulated by maternal mitochondrial DNA secreted from endometrial fluid-derived-EVs (50).

Proteome of uterine lavage-EVs may provide novel insights into biological processes critical for embryo development, implantation, and successful pregnancy (51). These EVs are dynamically regulated for their protein composition throughout menstrual cycle, transfer invasive properties and antioxidant function to trophectoderm cells (51). Moreover, these EVs carry proteins that regulate embryo implantation and predict WOI, thus highlighting their potential as a minimally invasive biomarker (51).

### Decidual Stromal Cell (DSC)-Derived EVs

Decidualization is the epithelioid transformation of endometrial stromal cells (eSC) during embryo implantation (23). Both endometrial stromal and epithelial cells can intake embryo-derived EVs (28), while DSCs-derived EVs can also be taken up by trophoblast cells and induce invasion through SMAD2/3-N-cadherin signaling pathway (32).

### Embryo-Derived EVs

Based on the latest research, bovine embryos secrete EVs with microRNA content according to embryonic competence and developmental stage. Similar to what was observed with EVs generated by other cell types, embryonic EVs are also involved in cellular signal transduction, and thereby regulating embryo implantation (21, 33). Supplementation of outgrowth embryo-derived EVs to the culture medium improved the development (52) and implantation capacity of preimplantation embryo (21). MVs produced by embryonic stem (ES) cells play an important role in stimulating trophoblast migration through the activation of FAK and c-Jun N-terminal kinase (JNK) (18). The injection of MVs isolated from ES cells into blastocysts increases the efficiency of embryo implantation (18). Human embryo-derived EVs have effects on endometrium by altering the expression of specific transcripts in endometrial epithelial cells (33). Interestingly, only good-prognosis embryos induced the observed effects while degenerated embryos failed to initiate any changes (33). Recently, an encouraging study reported that the size of EVs from culture medium of human embryos might be an alternative for evaluating their developmental competence (53). In the process of *in vitro* culture, the mean diameter of MVs/Exo from top quality embryos was higher (112.17 nm) than that of fair (108.02 nm) and poor quality embryos (102.78 nm) (P < 0.05) (53).

### Seminal Exosomes (SE)

Seminal fluid is not only the carrier for sperm delivery, but also a signaling agent that interact with female reproductive tissues to facilitate conception (54, 55). EVs are indispensable bioactive signaling factors in the crosstalk between seminal fluid and female reproductive system (34–36).

SEs of humans and pigs have been demonstrated to participate in the immuno-inflammatory responses of endometrium, and regulate uterine microenvironment related to embryo implantation through the changes of chemokines and cytokines (34, 35). In addition, a recent study has confirmed that SEs can promote the prolactin secretion of eSCs during WOI and enhance *in vitro* decidualization of human eSCs (36). This excited finding suggests that mechanisms by which SEs influence embryo implantation may be diverse.

### Oviductal EVs (oEVs)

Early embryonic development occurs in the oviduct, where an ideal microenvironment is provided by epithelial cells and by the oviductal fluid produced by these cells (56). OEVs are emerging as key players in the embryo-maternal interactions (37). During the process of bovine embryonic development, microRNA cargos of oEVs induce changes in embryonic gene expression which lead to a decrease of apoptosis of the embryonic cells and improve embryo viability which contribute to successful pregnancy (37, 38). The passage of gametes and the presence of embryo modulate microRNAs contents of oEVs (56), while the oviduct epithelial cell-derived exosomes improve the mitochondrial heath of *in vitro*-produced bovine embryos (39). Exosomes-treatment significantly upregulated the pyruvate dehydrogenase and glutamate dehydrogenase expression, required for metabolic fine-tuning of the TCA-cycle in the developing embryos (39). Melatonin was proved to be present in oviduct fluids and oEVs (40). The treatment of oEVs and melatonin enhances the *in vitro* development of embryo by regulating ROS and 5-mC levels (40).

### Mesenchymal Stem Cell (MSC) -Derived EVs

As an advanced therapeutic strategy, MSC therapy have been applied in many fields. MSCs play therapeutic and recovery roles not only through cell differentiation but also by secreting various paracrine signaling factors into the environment (46). However, low survival rate, immunological rejection and inevitable risk of tumor transformation limit its promise (44).

As a cell-free structure, MSC-derived EVs seem to be more promising due to its advantages of higher biological stability and easier perfusion into tissues (18, 41, 47, 57). As a new paradigm for endometrial-embryo crosstalk, EVs light the path to the research of embryo implantation and bring hope for the therapy of infertility in the future.

The proteomic characteristics of EVs derived from human endometrial mesenchymal stem cells (endMSC) are related to embryonic development and implantation (58). It was reported that endMSC-EVs exert an exogenous ROS scavenger activity during embryo culture (41), increase the developmental ability of IVF-derived embryos of elderly women, presumably by modulating the expression of antioxidant enzymes and promoting pluripotent activity (41). EndMSC-EVs enhance embryo quality reflected by a significant increase in total cell number per blastocyst and embryo hatching, and support angiogenesis, vascularization, immunoregulation, differentiation and tissue remodeling of the endometrium after embryo hatching (42, 43).

In rats with intrauterine adhesion (IUA), exosomes derived from adipose-derived mesenchymal stem cells (ADSC-exo) has an angiogenic effect on endometrial regeneration. Besides, ADSC-exo upregulates the expression of integrin and leukemia inhibitory factor (LIF) which are recognized as classic markers of endometrial receptivity (44). Bone marrow mesenchymal stem cell (BMSC)-derived exosomes may promote endometrial repair by the TGF-β1/Smad signaling pathway (45). Exosomes derived from umbilical cord-derived mesenchymal stem cell (UC-MSC) can also promote endometrial regeneration and fertility recovery through immunomodulation (47).

EVs from various tissues can promote implantation function by participating in intercellular communication, which is beneficial to embryo implantation process (shown as **Table 1**). However, it’s also important to note that these results are mostly obtained through *in vitro* tests and subsequent *in vivo* experiments are needed for further verification.

Given their fundamental role in regulating intercellular communication, it is not surprising that in some pathological contexts EVs can also play a negative role in embryo implantation. For example, endometrium-derived EVs from women with recurrent implantation failure (RIF) attenuate the growth and invasion of embryos (59). Therefore, EVs play a dual role in the process of embryo implantation, which may be attributed to the heterogeneity of EVs contents. Similar situations will be discussed in the following sections.

## RNA Cargos of EVs and Their Roles in Implantation

### MicroRNAs (miRNAs)

MiRNAs are a class of small non-coding RNAs that regulate gene expression either negatively by inhibition of translational repression or positively through the targeting of gene promoters (60). EV is one of the main carriers of miRNA *in vivo* (60, 61). The bilayer phospholipid membrane structure of EVs protects miRNAs from degradation and contributes to their stability (26). Evidences suggested that heterogeneous nuclear ribonucleoprotein C1 (hnRNPC1) may be involved in the internalization of endometrial miR-30d into exosomes to prepare for its subsequent incorporation into trophectoderm cells (62, 63). However, it is still unknown whether this protein is generally involved in miRNAs integration in EVs. Emerging evidences suggest the considerable role of EVs-derived miRNAs in embryo implantation events (27, 60). Among them, members of lethal-7, miR-30, miR-21 families are especially remarkable (64). Other miRNAs, such as miRNA-17-92 cluster, miR-29a, etc., have been proved to be closely related to embryo implantation process despite the temporary lack of EVs-related evidences (64, 65). There are 18 EVs-related references in this section.

#### Lethal-7 Family

The let-7 family, which has 12 members up to now, is important for cell development and proliferation inhibition (66). The diversity of let-7 is particularly noticeable for the establishment of endometrial receptivity and embryonic development (66–69).

During the whole event of embryo implantation, the expression level of let-7 family is dynamic (66, 67). In the preparation stage of embryo implantation, up-regulated let-7a/g in endometrial epithelial cells enhance endometrial receptivity by inhibiting the classical Wnt signaling pathway (68). It is well known that the acquisition of endometrial receptivity mainly depends on the precise control of estrogen and progesterone (70, 71). The level of miR-let-7a is regulated by estrogen and progesterone (68), which suggests that let-7 may be involved in the regulation of steroid hormones on embryo implantation (68). Interestingly, the expression level of let-7 family members (including let-7a/g) was significantly decreased in the developed blastocysts during implantation (67), which is contrary to what is found in endometrial epithelial cells.

A recent study has shown that placental exosome-derived bta-miR-499-5p is involved in the inhibition of NF-κB through the Lin28B/let-7 axis (72). As a member of the let-7 family, EVs-derived miR-98 regulates the maternal immune system of endometrium during the period of peri-implantation by regulating immune-related genes such as CTSC, IL6, CASP4 and IKBKE (69). The effect of let-7 on the regulation of immune response may be related to the apoptosis of endometrial cells and embryo implantation (69, 72), but further experiments are needed. A recent study has expanded the understanding of the regulatory role of let-7 family on embryo. Let-7 is a major factor that induces diapause in embryos. Let-7-containing EVs from uterine fluid induce mouse embryonic diapause by inhibiting c-myc/mTORC1 and mTORC2 signaling pathways (73, 74). Over-expression of EVs-derived let-7 potentially hamper trophoblast differentiation and the implantation capacity of embryo (73, 74).

#### MicroRNA-30 Family

As mentioned above, hnRNPC1 may be involved in the internalization of endometrial miR-30d into exosomes to prepare for its subsequent incorporation into trophectoderm cells (62, 63). During WOI, up-regulated miR-30 family in human endometrial epithelium is secreted into uterine fluid as exosome-associated molecule (75, 76). Hsa-miR-30d, secreted by human endometrium and taken up by the pre-implantation embryo, might modify its transcriptome, increase the adhesion rate *via* indirect overexpression of genes encoding for certain molecules involved in embryonic adhesion phenomenon, such as Itgb3, Itga7 and Cdh5 (75).

#### MicroRNA-21 Family

The miR-21 family has anti-apoptotic effects on many cellular biological processes, including regulating anti-apoptotic ability of preimplantation embryos (77). In pregnant mice, increased EVs and miR-21 in uterine luminal fluid regulate the growth of fertilized eggs and embryo development *via* apoptosis-related gene (Bax, Bcl-2, etc.) (78). Sus scrofa (ssc)-miR-21-5p regulates endometrial epithelial cell proliferation, apoptosis and migration *via* programmed cell death 4 (PDCD4)/AKT pathway (79).

Of note, the research progress of EVs-related miRNAs in embryo implantation is not limited to the above-mentioned families. During implantation, the expression of EVs-derived miRNAs, such as miR-34c-5p, miR-210 are significantly up-regulated in extracellular environment of uterine (80). Exosomal miR-100-5p not only promotes angiogenesis during implantation, but also activates both FAK and JNK to enhance the implant potency of trophoblasts (81). Moreover, despite the temporary lack of EVs-related evidences, numerous miRNAs have also been proved to play a regulatory role in embryo implantation (64, 65). It’s worthy to study whether EVs participate in the interaction between these miRNAs and embryo implantation. The specific discussion is as follows.

#### Other Implantation-Related MiRNAs

MiRNA-17-92 cluster is up-regulated at implantation site during WOI (64). Similarly, miR-29a is highly expressed in uterus to control implantation events (65), which may be achieved by inhibiting the apoptosis of eSC *via* targeting the pro-apoptotic factor genes Bak1, Bmf and the anti-apoptotic factor gene Bcl-w. As we all know, Cox-2-derived prostaglandins are critical to implantation, and a research have found that miR-101a and miR-199a regulate the implantation process by regulating Cox-2 post transcriptionally (82). MiR-31 targets immunoregulatory factors like FOXP3, CXCL12 and so on to achieve optimal endometrial receptivity through immunosuppression mechanisms (83).

However, miRNAs are complex and precise regulatory factor, and some miRNAs play negative regulatory roles during the process of embryo implantation. Higher expression of miR-200 family members is found in the serum of infertility and abortion women compared with that of healthy women (84). In vitro experiments have demonstrated that miR-200c inhibits proliferation and receptive ability of uterine epithelial cells *via* miR-200c/FUT4/α-1,3-fucosylation (LeY)/CD44/Wnt/β-catenin signal pathway (84). In addition, miR-661, which is specifically secreted by implantation-incompetent blastocysts, negatively regulates the adhesion of trophoblasts onto epithelium *via* PVRL1 *in vitro*, and may be involved in the breakdown of intercellular contact and loss of epithelial cell polarity in endometrium (85). Higher expression of miR-181b is also found in degenerated bovine embryos compared with fine blastocysts (86).

The above results (summarized in **Table 2**) strongly support the important role of miRNAs in embryo implantation, and the analysis of miRNAs describe a promising picture of the future in assisted reproduction. But the expression profiles of miRNAs are very variable in these studies mainly because of the complexity of miRNA signals, different species of experimental animals and individual heterogeneity (60). So, the emergence of new methodologies for miRNA extraction and quantification is urgently needed, and the role of EVs in embryo implantation needs to be discussed in categories. In the seventh section of this review, we will focus on the adverse effects of EVs on pathological embryo implantation.

**Table 2:** The reported miRNA cargos and their implication in embryo implantation.

MiRNA		Species	Site of action	Potential target genes/pathways in embryo-endometrial microenvironment	Possible effect on embryo implantation	Ref.
**Let-7 family**	**Let-7a/g**	Mice and human	Endometrium and embryo	Wnt/β-catenin-let-7 axis C-myc/mTORC1 and mTORC2 pathway	Promote implantation/ Induce embryonic diapause	(68, 73, 74)
	**Let-7g**	Mice	Blastocyst	Wnt/β-catenin-let-7 axis	Decrease embryo implantation	(66, 67)
	**MiR-98**	Cattle	Endometrium	Gene CTSC, IL6, CASP4 and IKBKE	Regulate the maternal immune system of endometrium	(69)
**MiR-30d**		Human	Embryo	Gene Itgb3, Itga7 and Cdh5	Promote embryo adhesion	(75)
**MiR-21**		Mice	Embryo	Gene Bax, Bcl-2, etc.	Promote embryonic development	(77, 78)
		Sus scrofa	Endometrial epithelium	PDCD4/AKT pathway	Regulates the function of endometrial epithelium	(79)
**MiR-29a**		Rat	Endometrium	Gene Bak1, Bmf and Bcl-w	Inhibit the apoptosis of endometrial stromal cells	(65)
**MiR-101a and miR-199a**		Mice	Uterus	Cox-2	The exact mechanism remains unknown	(82)
**MiR-31**		Human	Endometrium	FOXP3, CXCL12, etc.	Promote endometrial receptivity	(83)
**MiR-100-5p**		Ishikawa cell lines	Trophoblast	FAK or JNK signaling	Promote migration and invasion	(81)
**MiR-200**		Human	Endometrial cell	MiR-200c/FUT4/LeY/CD44/Wnt/β-catenin pathway	Inhibit proliferation and receptive ability	(84)
**MiR-661**		Human	Endometrial epithelium	Gene PVRL1	Decrease the adhesion of trophoblasts onto epithelium	(85)

#### Long Non-Coding RNAs (lncRNAs)

As the most heterogeneous class of non-protein-coding RNA, with lengths ranging from 200 to 100,000 nt, lncRNAs are involved in almost all biological processes (87). EVs may be involved in the process of lncRNAs avoiding the degradation of ubiquitous RNase in body fluids, so as to reach target cells and play a regulatory role (87, 88). So far, there are few studies on EVs-derived lncRNAs in embryo implantation, but the research progress of EVs-derived lncRNAs in some endometrium-related diseases has brought clues for future research. EVs from endometriosis women are characterized by a unique miRNA-lncRNA signature (89), which may affect endometrial receptivity during WOI result in implantation failure (90). Decidualization and angiogenesis are typical changes in the endometrium during implantation, and antisense hypoxia-inducible factor (aHIF) is a well-known angiogenesis-related lncRNA (87). Endometriotic cyst stromal cells-derived exosomal aHIF induces angiogenesis by regulating angiogenesis-related genes in human umbilical vein endothelial cells (87). Some other specific lncRNAs are associated with endometrial physiopathology and embryo implantation (91, 92). LIF is a kind of cytokine secreted by endometrial glands and plays an amazingly important role in embryo implantation during WOI (93, 94). As a competing endogenous RNA for miR‐15b, lncRNA882 regulates LIF by sponging miR‐15b in endometrial epithelium cells of dairy goat (95). (There are 3 EVs-related references in this section.)

## Protein Cargos of EVs and Their Roles in Implantation

Proteomic profiling of endometrium has revealed that endometrial exosomes contain a number of unique exosomal proteins not previously identified in exosomes from any other tissues (29). These endometrium-derived exosomal proteins are primarily regulated by estrogen and progesterone during menstrual cycle (29), which is consistent with the establishment of endometrial receptivity (70, 71). Importantly, proteomic changes in human trophectoderm function are demonstrated after endometrial EVs are internalized by human trophectoderm cells, which probably due to the transfer of EV protein cargos (26). Therefore, the expression differences of exosomal protein during the various stages of menstrual cycle may be closely related to embryo implantation. There are 5 EVs-related references in Section 6.

Among the numerous exosomal proteins, matrix metalloproteinase (MMP), which may have potential roles in embryo-maternal crosstalk during implantation has attracted much attention (22, 29). MMPs are widely expressed on maternal-fetal interface, responsible for extracellular matrix degradation and regulated by tissue inhibitors of MMPs (TIMPs) (96, 97). Different expression of MMP2, MMP14, and TIMP2 is observed in endometrium during various phases of the estrous cycle (98). MMPs contribute to the spatial and temporal matrix remodeling in bovine endometrium and may be related to the invasive ability of trophoblast cell (96). Some MMPs are relevant to endometrial angiogenesis (99), which is essential for vascularized receptive endometrium.

HLA-G is a key molecule in the process of embryo implantation, avoids the maternal immune rejection of the embryo by regulating the maternal-fetal immune response, and mediates communication with target cells in a variety of ways (100). The expression of HLA-G protein at the maternal-fetal interface is critical to the success of pregnancy, and HLA-G can be secreted with EVs (100).

So far, researches on the role of EVs-derived proteins in embryo implantation process are still not in-depth. In vitro experiments have discovered that 254 and 126 proteins are uniquely enriched in endometrial-derived exosomes during the proliferation and receiving phases, respectively (29). Whether these numerous and diverse proteins can provide appropriate conditions for embryo needs further explosion.

## EVs and Embryo Implantation: A New Viewpoint of Reproductive Diseases

It is widely accepted that women with a history of reproductive diseases such as recurrent implantation failure (RIF), polycystic ovary syndrome (PCOS), and endometriosis, are associated with future impaired embryo implantation (101–105). EVs-based intercellular communication plays an important role in female reproductive microenvironments and is involved in the pathogenesis of these disorders (59, 89, 106–109). The regulatory disorders caused by abnormal EVs may provide a new viewpoint of implantation failure in women with reproductive diseases (summarized in **Table 3**). There are 9 EVs-related references in Section 7 (3 for endometriosis; 4 for PCOS; 2 for RIF).

**Table 3:** EVs in reproductive diseases and their potential roles in embryo implantation disorders.

Reproductive diseases	The potential role of EVs in embryo implantation disorders	Ref.
**Endometriosis**
	Affect the expression of endometrial receptivity marker molecules, such as LIF, HOXA10	(110)
	Angiogenesis	(89, 108)
	Immuno-inflammatory responses	(89)
**PCOS**
	Disturbances of ovarian steroidogenesis	(106)
	Abnormal estrogen secretion	(107)
	Affect inflammation, ROS metabolic process, cell migration and proliferation	(106)
**RIF**
	Inhibit blastocyst formation	(59)
	Inhibit the proliferation, migration, and invasion of trophoblast cells	(59, 109)

### EVs in Endometriosis

Endometriosis is defined by the presence of viable endometrial tissue outside the uterine cavity (103). A number of implantation markers such as αvβ3 integrin, LIF, homeobox A10 (HOXA10) and HOXA11 are aberrantly expressed in patients with endometriosis and may contribute to infertility in some women with endometriosis (103). Endometrial defects and defective endometrial-embryo cross-talk are both proposed mechanisms of implantation failure in endometriosis (103, 111). In recent years, the role of EVs in the process of implantation failure in endometriosis women is attracting increasing attention, and changes in the expression of certain proteins, lncRNAs and mRNAs may affect endometrial receptivity in rats with endometriosis during WOI, probably resulting in implantation failure of the embryo (89, 90, 108, 110).

EVs, including exosomes obtained from tissues (including endometrium, eutopic and ectopic endometriotic lesions, peritoneal fluid) and plasma samples of endometriosis women have unique miRNA-lncRNA characteristics (89). Forty-nine differentially expressed miRNAs are identified in eutopic endometrial stromal cells exosomes compared with that of normal endometrial stromal cells exosomes, and 12 miRNAs are predicted to target HOXA10, which is also a hopeful predictor of endometrial receptivity (112, 113), and/or the LIF 3’ untranslated region (110). In addition, higher levels of angiogenic and inflammatory cytokines are present in the human umbilical vein endothelial cells cocultured with the endometriotic epithelial exosomes (89, 108). The proinflammatory microenvironment stimulated by eutopic endometriotic lesions-derived EVs may also responsible for the disorders of embryo implantation in endometriosis (89, 108).

### EVs in PCOS

PCOS, one of the most common endocrine disorders in women, affects 8-13% of women of reproductive age (114). Nowadays, sufficient evidences have proved that hormonal disturbances as well as metabolic changes in PCOS women can both affect endometrial receptivity and embryo implantation (101, 102).

At gene level, there is a differential gene expression in endometrium of PCOS detected by microarray evaluation (115, 116). It is noteworthy that most of these genes are involved in steroid hormone synthesis, inflammation and oxidative stress, which are indispensable for the establishment of endometrial receptivity (116). The proteome pattern of endometrium during WOI in PCOS women is significantly different with that of normal female (117, 118). These differences in transcription, post-transcriptional modification and translation are often related to metabolism, cell cycle, DNA repair, apoptosis and signal transduction (116), and may consequently cause impaired endometrial receptivity in PCOS women. But the specific mechanisms are still unknown. Researches on EVs may provide new insights into the further understanding of implantation failure in PCOS.

RNA sequencing microarray and proteomic analysis have shown differentially expressed small RNAs, circRNAs, and 86 proteins in follicular fluid exosomes of PCOS women (106, 107, 119, 120). The alterations of the proteomic profile of PCOS women are related to the inflammation, reactive oxygen species metabolic process, cell migration and proliferation (106), which are closely related to embryo implantation. S100‐A9 protein in exosomes derived from follicular fluid promotes inflammation and causes disorders of ovarian steroidogenesis *via* activation of NF‐κB pathway (106). The down-regulated exosomal circLDLR in follicle fluid of PCOS women is proposed to involve in abnormal estrogen secretion as well (107).

Chronic inflammation is an acknowledged cause of endometrial physiological dysfunction (121, 122). Excessive inflammation results in the adverse pregnancy outcomes in PCOS and endometriosis. The articles above (106, 107) specialize in local inflammation and steroidogenesis of the ovary, and the disorders of ovarian steroidogenesis and abnormal inflammatory state caused by follicle fluid exosomal cargos may also be the potential mechanisms of the disorders of embryo implantation in PCOS.

### EVs in RIF

RIF refers to the repeated transfer of morphologically good embryos to a normal uterus without achieving successful implantation and a clinical pregnancy (123). Traditionally, the failure to achieve a clinical pregnancy after transfer of at least four good-quality embryos in a minimum of three fresh or frozen cycles in a woman under the age of 40 years is defined as RIF (105). The etiologies and pathogenesis of RIF is unknown, and clarifying the mechanisms of EVs in RIF may provide benefits to the treatments of embryo implantation disorders.

Altered miRNA profiles in RIF-EVs might be involved in the pathogenesis of RIF. The up-regulated miR-1246 and miR-1290 in the RIF-EVs may indicate the inadequate endometrial receptivity of RIF women (124). In vitro experimental studies have demonstrated that endometrial EVs from RIF women attenuate embryonic development and implantation capacity by inhibiting blastocyst formation, decreasing the total cell number of embryos as well as inhibiting the proliferation, migration, and invasion of trophoblast cells (59, 109).

## Prospects and Conclusions

**Figure 2:**
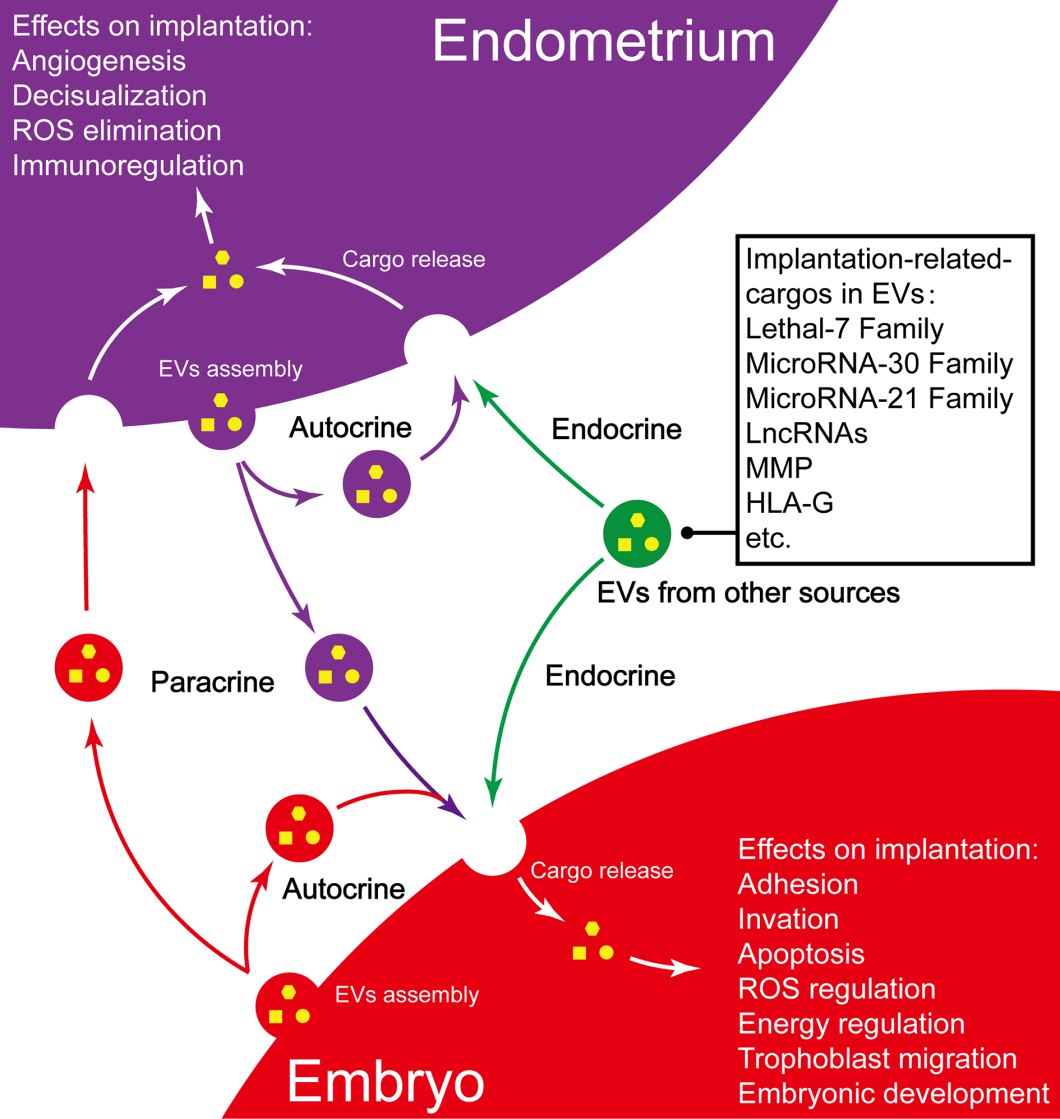
Summary of EVs and their implication in embryo implantation. Purple vesicles, endometrium-derived EV; Red vesicles, embryo-derived EVs; Green vesicles, EVs from other sources.

In conclusion, as one of the important communication mechanisms that modulate blastocyst and endometrial functions, EVs play an important role in promoting embryo implantation, which mainly owes to EVs’s unique ability in transferring heterogeneous cargos (summarized in **Figure 2**) (125). Abnormal EVs play a negative role in some pathological conditions.

EVs have shown great potential as molecular biomarkers in diagnosis, prognosis and also as possible therapeutic targets. Currently, endometrial dysfunction is the biggest obstacle to the treatment of embryo implantation disorders. EVs derived from serum and uterine fluid may be used, as a noninvasive and accurate marker of endometrial station, to identify women with implantation defects, demonstrate the optimal timing for embryo transfer, and replace the traditional endometrial biopsy (83, 126, 127). EVs-based preparation may be a promising approach to endometrial regeneration and improving pregnancy outcomes (128).

But the precise mechanisms of EVs regulating embryo implantation have not been elucidated enough. The following points deserve attention and improvement particularly: 1. the data of EVs cargos are mainly derived from immortalized or long-term passaged cell lines. 2. there is still no golden standard of EVs separation, concentration and purification, and no acknowledged nomenclature of subclassing EVs with various biophysical properties. 3. the experimental data of effects of EVs in embryo implantation is largely based on *in vitro* trophoblast adhesion/invasion assays, but the corresponding *in vivo* evidence has not been rigorously established. 4. current understanding of EVs has been limited to protein and RNA cargos, but the role of EVs-derived DNA and lipid molecules in embryo implantation remains unknown. So further exploration of the effects of EVs and their cargos on embryo implantation is still needed.

## Author Contributions

N-XJ wrote the manuscript. X-LL designed the topic and critically revised our work. All authors contributed to the article and approved the submitted version.

## Funding

This study was funded by Natural Science Foundation from Science and Technology Commission of Shanghai Municipality (grant No. 17ZR1403100 to X-LL).

## Conflict of Interest

The authors declare that the research was conducted in the absence of any commercial or financial relationships that could be construed as a potential conflict of interest.
